# MiR-217 regulates autophagy through OPG/RANKL/RANK in giant cell tumors

**DOI:** 10.1186/s13018-023-03826-1

**Published:** 2023-05-10

**Authors:** Chenyang Meng, Boyong Jiang, Wanlin Liu, Lianjuan Wang, Zhenqun Zhao, Rui Bai, Yan Zhao

**Affiliations:** 1 The Second Affiliated Hospital of Inner Mongolia Medical University, Hohhot, 010030 China; 2 Bao Tou Medical College, Baotou, 014040 China

**Keywords:** Giant cell tumors (GCT), miR-217, OPG/RANKL/RANK signal pathway, Autophagy

## Abstract

**Background:**

Increasing evidence suggests that microRNAs (miRNAs) play a crucial role in cancer development and progression. Our previous study showed remarkably lower levels of miR-217 in GCT cells and tissues, and miR-217 re-expression inhibited the occurrence and development of GCT in vitro; however, the associated mechanisms remain unknown. Thus, this study aimed to explore the mechanisms underlying the proliferation inhibitory effect of miR-217 in GCT cells.

**Methods:**

The proliferative potential of the GCT cells was measured with an MTT assay and BrdU straining. Changes in GCT cell migration and invasion was assessed by a transwell assay. Finally, Western blot and RT-PCR assays were employed to evaluate OPG/RANKL/RANK signaling pathway-related protein expression.

**Results:**

The excessive upregulation of miR-217 markedly suppressed GCT cell proliferation and tumorigenesis both in vitro and in vivo*.* miR-217 overexpression could inhibit the OPG/RANKL/RANK signaling pathway in vitro and in vivo. Furthermore, ALP activity was significantly decreased in GCT cells following miR-217 treatment. Importantly, miR-217 could inhibit autophagy-related protein expression and autophagosome/autolysosome formation in GCT cells and tissues.

**Conclusion:**

These results suggest that miR-217 upregulation could inhibit the occurrence and development of GCT by blocking autophagy. These findings offer an effective therapeutic target to improve the survival rates of patients with CGT in the future.

## Introduction

Giant cell tumor (GCT) of the bone is a benign tumor and it is most commonly diagnosed in adults between the ages of 20 to 40 years [1, 2]. GCT cells are often associated with potential invasion, local recurrence, and occasionally distant metastasis [3]. Furthermore, GCT cells grow in an expansive manner and can easily penetrate the cortex of the bone or even cause pathological fractures [4]. Surgery represents the mainstay of GCT treatment; however, wide resection requires complex reconstruction of the adjacent joints, which increases the rate of surgical complications and disabilities [5, 6]. Therefore, further investigation into the molecular mechanisms underlying GCT occurrence and development will aid in the identification of novel therapeutic targets and strategies.

Osteoprotegerin (OPG) is a member of the TNF receptor superfamily which bonds to the receptor activators of nuclear factor κB ligand (RANKL) and TNF-related apoptosis-inducing ligand (TRAIL) [7, 8]. Furthermore, OPG has been shown to inhibit RANKL and RANK binding, reduce osteoclast activity, and impede bone resorption [9]. These findings suggest that the OPG/RANKL/RANK system plays a critical role in maintaining bone balance, and represents the physiological basis for regulating osteoclast formation [10, 11]. Moreover, recently published studies have demonstrated that the OPG/RANKL/RANK system is deeply involved in the occurrence and development of various cancers, particularly bone metastases in various cancers [12, 13]. Therefore, the OPG/RANKL/RANK system has attracted increased attention as a potential therapeutic target in cancer.

Autophagy is a cellular catabolic pathway through which cells eliminate misfolded intracellular proteins and damaged organelles through lysosomal degradation to recycle their nutrients [14]. Importantly, under nutrient- or growth factor-deficient conditions, autophagy is essential for the maintenance of energy production for cell survival [15]. Additionally, autophagy has been confirmed to be an adaptation mechanism for protecting cells against anticancer treatment, and the inhibition of autophagy can increase the likelihood that cancer cells more likely will undergo apoptosis in response to anticancer agent treatment [16, 17]. Furthermore, basal autophagy could also promote tumor survival and progression, making it a potential therapeutic target for cancer [18]. Although there have been some reports regarding the role of autophagy in GCT, the relationship between the OPG/RANKL/RANK signaling pathway and autophagy in GCT are poorly understood.

MicroRNAs (miRNAs) are a class of small non-coding RNAs (19–25 nucleotides) that regulate the genome at both the transcriptional and post-transcriptional levels [19]. Currently, hundreds of miRNAs have been identified in eukaryotes, which can control a variety of biological processes, including cell proliferation, apoptosis, angiogenesis, invasion, metastasis, the immune response, and differentiation [20–22]. Importantly, more studies have associated miRNA dysregulation with tumorigenesis and stress adaptation [23]. Therefore, miRNAs can be considered as potential therapeutic targets of cancer treatment, and they hold great potential for explaining the mechanisms of cancer occurrence and development. In this study, we identified an miRNA termed miR-217, and found that it was abnormally expressed in GCT. However, the specific role and function of miR-217 in GCT occurrence and development remains poorly understand. Therefore, this study aimed to clarify the role and function of miR-217 in GCT, the findings of which will contribute to understanding GCT and providing an excellent target for GCT treatment.

## Materials and methods

### Cell cultures

GCT0404 cells were obtained from the American Type Culture Collection (ATCC) (Manassas, VA, USA). Cells were cultured in RPMI Medium 1640 (Gibco, Gaithersburg, MD, USA) supplemented with 10% Fetal Bovine Serum (FBS) (TBD, Biotechnology Development, Tianjin, P.R.C.) and 1% penicillin/streptomycin in a humidified incubator at 37 °C and 5% CO_2_.

### MiRNA and shRNA transfection

MiR-217 mimics, mimic negative control, miR-217 inhibitor, or inhibitor negative control oligonucleotides (Guangzhou RiboBio, Guangzhou, China) were transfected into GCT0404 cells using lipofectamine 3000 transfection reagents (Beyotime Biotechnology, Nanjing, P.R.C.) with a final concentration of 100 nM. ShRNAs for OPG were also transfected into GCT0404 cells using lipofectamine 3000 transfection reagents at a concentration of 50 nM. The sequences of the mimics and inhibitor used for miR-217 overexpression and knockdown, as well as the shRNA sequence used for OPG knockdown were listed as follows: miR-217 inhibitor: UCCAAUCAGUUCCUGAUGCAGUA and miR-217-mimic: UACUGCAUCAGGAACUGAUUGGA, respectively. Scramble mimics: UUCUCCGAACGUGUCACGUTT and inhibitor NC: CAGUACUUUUGUGUAGUACAA were used as controls. OPG siRNA: TGATCTTCTTGACTATATCTTGGTC and NC siRNA: GGATTCCTTGTATATTGCTCTCTAT were used to construct sh-OGP and sh-NC by GenePharma (Shanghai, China). RT-PCR and Western blot assays were performed to confirm the transfection efficiencies of miRNAs or shRNA,

### Cell proliferation assay

TGCT0404 cells were cultured in RPMI-1640 medium containing 10% fetal bovine serum, 100 U/mL penicillin, 100 µg/mL streptomycin at 37 °C and 5% CO2. When the cells grew to 80–90%, the medium was removed and washed 1–2 times with PBS. After the PBS was removed, 1 mL trypsin was added for digestion for 1–3 min. Next, 3 mL complete medium was added to neutralize trypsin to terminate digestion, and the digested cells were transferred to a 15 mL centrifuge tube for centrifugation at 1000 rpm for 5 min. After the supernatant was discarded, the cells were re-suspended in 3 mL medium, and passed 1:3 into a culture dish. GCT0404 cells that grew to 80%-90% in the culture dish were digested with trypsin, centrifuged at 1000 rpm, the supernatant was removed, and complete medium was added to re-suspend the cells. The cells were seeded into six-well plates with 1 × 10^6^ cells per well, such that the cell density during transfection reached 30–50%. After cell adherence, the cells were infected with an miR-217 overexpressing virus and miR-217 under expressing virus and their corresponding NC. Culture plates were placed in a CO2 incubator at 37 °C for 24–96 h. The cells were digested with pancreatic enzymes, centrifuged at 1000 rpm, the supernatant was removed, and the cells were resuspended in complete medium. The cells were counted in a blood cell counting plate with 5000 cells per well in the 96-well plate. Each 96-well plate was planted with three multiple pores and a total of six plates were planted. After cell adhesion, 10 µL MTT (5 mg/mL) was added to the medium and further cultured in an incubator for approximately 3 h. The medium was removed, 150 µL DMSO was added to each well, and the absorbance was measured at 570 nm.

### Colony formation assay

Cells transfected with the aforementioned miRNAs were seeded into six-well plates (2000 cells per well). After 2 weeks of cell culture, cells were fixed with 4% paraformaldehyde and stained with crystal violet. Finally, the colonies (> 50 cells) were imaged and counted by a digital camera (Canon, Tokyo, Japan).

### ALP activity detection

Cells were transfected with the aforementioned miRNAs and cultured for the indicated time periods. Next, the cells were washed in ice-clod PBS for twice and lysed with RIPA Lysis Buffer (Beyotime Biotechnology, Nanjing, P.R.C.) for 30 min. The alkaline phosphatase (ALP) activity of cells was determined using an ALP activity assay kit (Nanjing Jiancheng Bioengineering Institute, Nanjing, P.R.C.). According to the manufacturer's instructions, the absorbance was measured with a microplate reader at 405 nm.

### Acridine orange staining

1. GDT-0404 cells were cultured in RPMI1640 medium containing 10% fetal bovine serum, 100 U/mL penicillin, 100 µg/mL streptomycin in a cell incubator containing 5%CO2 at 37 °C.
  Cell passage culture: when the cells grew to 80–90%, the medium was removed and washed 1–2 times with PBS. After the PBS was removed, 1 mL trypsin was added for digestion for 1–3 min, and 3 mL complete medium was added to neutralize trypsin to terminate digestion. The digested cells were transferred to a 15 mL centrifuge tube for centrifugation at 1000 rpm for 5 min. After the supernatant was poured out, the cells were re-suspended in 3 mL medium, and passed 1:3 into a culture dish.
2. GDT-0404 cells that reached 80–90% confluency in the petri dish were digested with trypsin, centrifuged at 1000 rpm, supernatant was removed, complete medium was added to re-suspension cells, and cells were planted in six-well plates with 1 × 10^6^ cells per well.
3. After cell adhesion, the cells were transfected with NC mimic, inhibitor NC, and an inhibitor using liposome. A) Dilution: 50 µL serum-free medium. Opti-MEM was used to dilute 50 nM NC, mimic, inhibitor NC, inhibitor, mixed gently, incubated at room temperature for 5 min; b) Dilution lipo3000: 3 µL lipo3000 was diluted with 50 µL serum-free medium Opti-MEM was gently mixed and incubated at room temperature for 5 min; a and b were gently mixed, incubated at room temperature for 20 min, then added to six-well plate (each well of six-well plate contained 100 µL liposome transfection mixture), and cultured in an incubator. After 6 h culture, complete culture medium was replaced. The petri dish was place in an incubator to grow.
4. After 24 h cell culture, 100 nM rapamycin was added to the medium, gently mixed, and cultured for 24 h.
5. The cells were removed from the incubator, the medium was removed, the cells were digested with pancreatic enzymes, and pre-cooled PBS was added to gently wash the cells twice.
6. The cells were transferred into a flow tube and placed on ice.
7. A total of 0.4 mL cold cell breaker was added and placed on ice for 15 s.
8. A total of 1.2 mL cold acridine orange dye was added and placed on ice, away from light.
9. The fluorescence of cells was detected and recorded within 2–10 min after adding acridine orange solution.

### Transmission electron microscopy

Cells transfected with miRNA were fixed in 2.5% glutaraldehyde overnight at 4 °C. After washing in buffer, the cells were washed in PBS, followed by fixation in 1% OsO_4_ with 1% K_3_Fe(CN)_6_ for 1 h at 4 °C. After dehydrating in graded ethanol, infiltrating in tert-butanol, and embedding in epoxy resin, the cells were sliced into ultra-thin sections (60-nm-thick) and stained again with 2% uranyl acetate and 1% lead citrate. Finally, the sections were examined using a Philips CM100 electron microscope at 110 kV. Images were digitally recorded using a Hamamatsu ORCA-HR digital camera system.

### Tumor xenograft model

All animals were cared for in accordance with the Animal Welfare Act guidelines under an animal protocol approved by the Inner Mongolia Medical University Animal Care and Use Committee. After GCT0404 cells were transfected with either the miR-217 mimic or miR-217 inhibitor, they were mixed with 50% Matrigel (BD Biosciences, San Jose, USA) in 100 μL PBS. Next, the cell suspension was subcutaneously injected into four-week-old male BALB/c nude mice (5 × 10^6^ cells/mice). The mice were breed for six weeks under normal conditions and the tumor size was measured every four days using a digital caliper. The tumor volumes were determined using the formula: tumor volume = (length × width^2^)/2. The average tumor volume in each group was calculated and tumor growth curves were drawn accordingly. By the end of the experiment, all mice were euthanized by an overdose of CO_2_ exposure, and all tumors were collected and measured.

### Real-time PCR (RT-PCR)

Using the standard process, the total RNA was extracted using the Trizol method. RT-PCR was performed using SYBR Green Master mix according to the supplier protocols (Invitrogen, Carlsbad, CA, USA). The relative expression levels of miRNA and mRNA transcripts were calculated using the 2^−ΔΔCt^ method, and the level of GADPH expression was used to compare the relative level of mRNA expression in each sample. Primers synthesized by Genechem (Shanghai, China) are shown in Table 1.

**Table 1:** Primers used for real-time PCR

	Primer sequences	
miR-217	(F)	5′-AACAGTGTACTGCATCAGGAAC -3′
	(R)	5′-GTCGTATCCAGTGCAGGGT -3′
U6	(F)	5′-GTGCTCGCTTCGGCAGCACATATAC -3′
	(R)	5′-AAAAATATGGAACGCTTCACGAATTTG -3′
OCN	(F)	5′-TGCAGAGTCCAGCAAAGG -3′
	(R)	5′-CCCAGCCATTGATACAGGTAG -3′
RUNX2	(F)	5′-ACCTTGACCATAACCGTC TTC -3′
	(R)	5′-GCTTCTGTCTGTGCCTTCT -3′
OPG	(F)	5′-CATTCTTCAGGTTTGCTGTTCC -3′
	(R)	5′-CTCTCTACACTCTCTGCGTTTAC -3′
RANK	(F)	5′-CCATGTACCAGTGAGAAGCATTA -3′
	(R)	5′-CTGTCAGAGGTAGTAGTGCATTTAG -3′
RANKL	(F)	5′-GTGGATGGCTCATGGTTAGAT -3′
	(R)	5′-GAGGACAGACTCACTTTATGGG -3′
ATG5	(F)	5′-CTTCTGCACTGTCCATCTAAGG -3′
	(R)	5′-ATCCAGAGTTGCTTGTGATCTT -3′
ATG7	(F)	5′-TTGCCCACAGCATCATCTT -3′
	(R)	5′-ATGCCTCCTTTCTGGTTCTTT -3′
Beclin-1	(F)	5′-GCAGCTGGATAAGCTGAAGA -3′
	(R)	5′-CGACCCAGCCTGAAGTTATT -3′
LC3	(F)	5′-GTCCTGGACAAGACCAAGTTT -3′
	(R)	5′-GTTCACCAGCAGGAAGAAGG -3′
GAPDH	(F)	5′-CAGGGCTGCTTTTAACTCTGGTAA -3′
	(R)	5′-GGGTGGAATCATATTGGAACATGT -3′

### Western blotting

Cells were collected and mixed in RIPA Lysis Buffer for 30 min at 4 °C followed centrifuged at 12,000×*g* at 4 °C for 15 min. The supernatant was collected to quantify the concentration of total protein using a BCA Protein Assay kit (Beyotime Biotechnology, Nanjing, P.R.C.). An equal amount of the total cell lysates were separated by 10% sodium dodecyl sulfate–polyacrylamide gel electrophoresis (SDS-PAGE), and transferred onto polyvinylidene fluoride (PVDF) membranes (Millipore Corporation, USA). The membranes were probed with primary antibodies (1:300–1:1000) at 4 °C overnight and subsequently incubated with a horseradish peroxidase (HRP)-conjugated secondary antibody at 37 °C for 2 h. The level of target protein expression was visualized using an enhanced ECL kit (Beyotime Biotechnology, Nanjing, P.R.C.). Densitometry analysis was performed using Image J 1.44 software.

### Immunohistochemistry histochemistry

The collected tumors were fixed in 4% paraformaldehyde, embedded in paraffin, and sliced into 4-μm-thick sections using a microtome. After removing paraffin wax with xylene, the tumor sections were incubated with the primary antibody, and subsequently incubated with a secondary antibody labeled with HRP. The staining was visualized by 3, 3-diaminobenzidine (DAB) and Harris hematoxylin and then analyzed under an Olympus microscope.

### Statistical analyses

All of the results were presented as the means ± standard deviation (SD) of three independent experiments unless stated otherwise. Comparisons of the means among multiple groups were accomplished by a one-way analysis of variance (ANOVA) and a multiple range least significant difference (LSD) was used for inter-group comparisons. *P* values < 0.05 were considered statistically significant. All statistical analyses were performed with SPSS 22.0.

## Results

### MiR-217 suppresses the proliferation of GCT cells

**Fig. 1:**
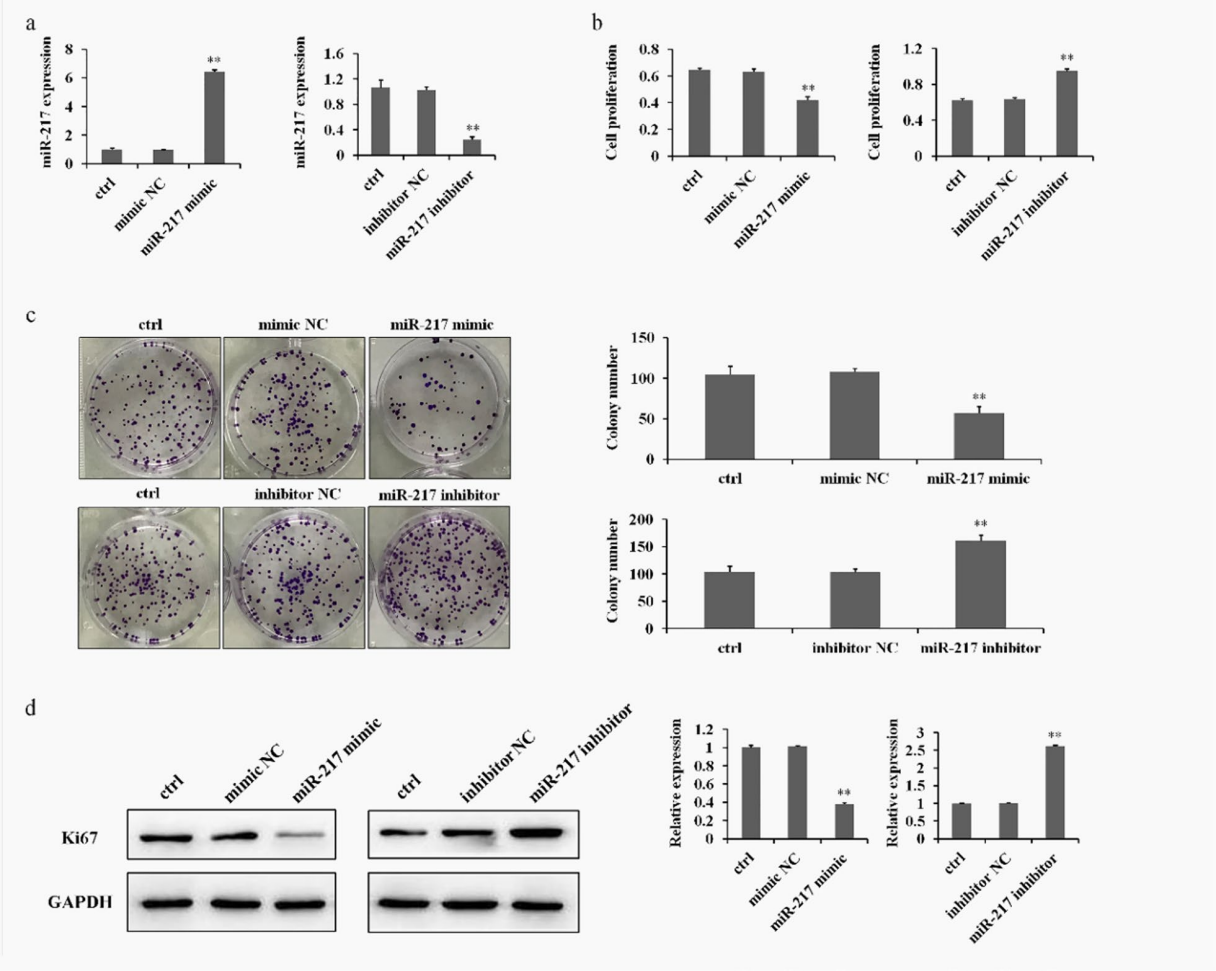
MiR-217 suppresses GCT cell proliferation. **a** The levels of miR-217 expression were detected in miR-217 mimic or miR-217 inhibitor-treated GCT0404 cells by RT-PCR. **b**, **c** MTT and colony formation assay for the cellular proliferation of GCT0404 cells treated with miR-217 mimics or miR-217 inhibitor. **d** Western blot showing the changes in Ki67 expression (molecular weight 359 kDa) in miR-217 mimic or miR-217 inhibitor-treated GCT0404 cells. The molecular weight of GAPDH is 36 kDa. **P < 0.01 versus control

It has been well-established that miRNAs are involved in a series of biological functions [19]. To study the roles played by miR-217 in GCT, we performed an MTT assay and colony formation assay to evaluate the effect of miR-217 on the proliferation of GCT0404 cells. As shown in Fig. 1a, b, treatment with the miR-217 mimics increased miR-217 expression in GCT0404 cells, and significantly inhibited the proliferation of GCT0404 cells compared to that of the control group. In contrast, treatment with the miR-217 inhibitor significantly promoted GCT0404 cells proliferation (Fig. 1b). Additionally, treatment with the miR-217 mimics suppressed GCT cell colony formation, whereas treatment with the miR-217 inhibitor increased the number of colonies in GCT cells (Fig. 1c).

The Ki67 protein is a cellular marker for tumor proliferation, and presents during all active phases of the cell cycle [24]. The Western blot results from western blots showed that the expression of Ki67 was significantly decreased by miR-217 mimics; however, treatment with the miR-217 inhibitor up-regulated Ki67 expression in GCT0404 cells (Fig. 1d). Together, these results suggest that miR-217 is a key factor in regulating GCT cell growth, and can therefore affect GCT occurrence and development through regulating Ki67.

### MiR-217 inhibited the OPG/RANKL/RANK signaling pathway in GCT cells

**Fig. 2:**
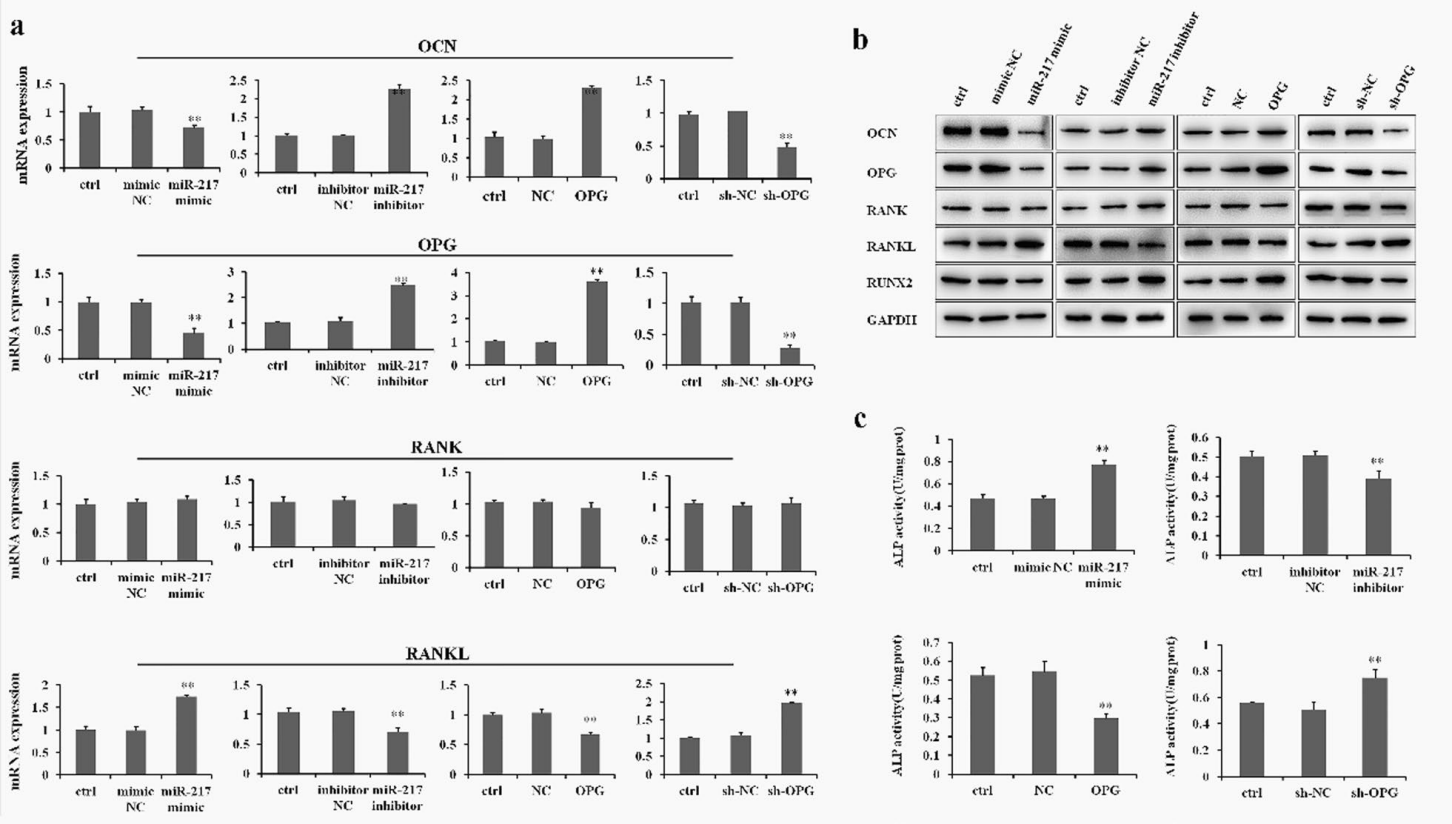
MiR-217 inhibits the OPG/RANKL/RANK signaling pathway in GCT cells. **a**, **b** The levels of OCN expression (molecular weight 37 kDa), OPG (molecular weight 46 kDa), RANK (molecular weight 55 kDa), and RANKL (molcular weight 35–45 kDa) were detected in GCT0404 cells by RT-PCR and Western blot. The molecular weight of RUNX2 is 55–62 kDa. **c** Detection of ALP activity in GCT0404 cells by miR-217 or OPG treatment. **P < 0.01 versus control

The OPG/RANKL/RANK signaling pathway represents the physiological basis for regulating osteoclast formation [11]. RT-PCR and Western blot results have shown that miR-217 overexpression via transfection with miR-217 mimics could remarkably decrease the expression of OCN and OPG, but increase RANKL expression in GCT0404 cells (Fig. 2a, b,). In contrast, the expression of OCN and OPG were found to be significantly increased, and RANKL expression was decreased following treatment with an miR-217 inhibitor in GCT0404 cells (Fig. 2a, b).

Furthermore, OPG overexpression could also induce an increase in OCN expression and down-regulate RANKL expression in GCT0404 cells (Fig. 2a, b). Interestingly, neither miR-217 nor OPG affected the level of RANK expression in GCT0404 cells (Fig. 2a, b). In addition, RUNX2 expression was significantly decreased by treatment with the miR-217 mimics and OPG (Fig. 2b). Moreover, ALP activity, which reflects osteoblastic activity and bone turnover, is a well-known bone biomarker [25]. Figure 2c shows that ALP activity could be significantly increased by treatment with miR-217 mimics and sh-OPG, respectively. In contrast, both treatment with the miR-217 inhibitor and OPG overexpression were associated with decreased ALP activity in GCT0404 cells (Fig. 2c).

### MiR-217 inhibits autophagy in GCT cells

**Fig. 3:**
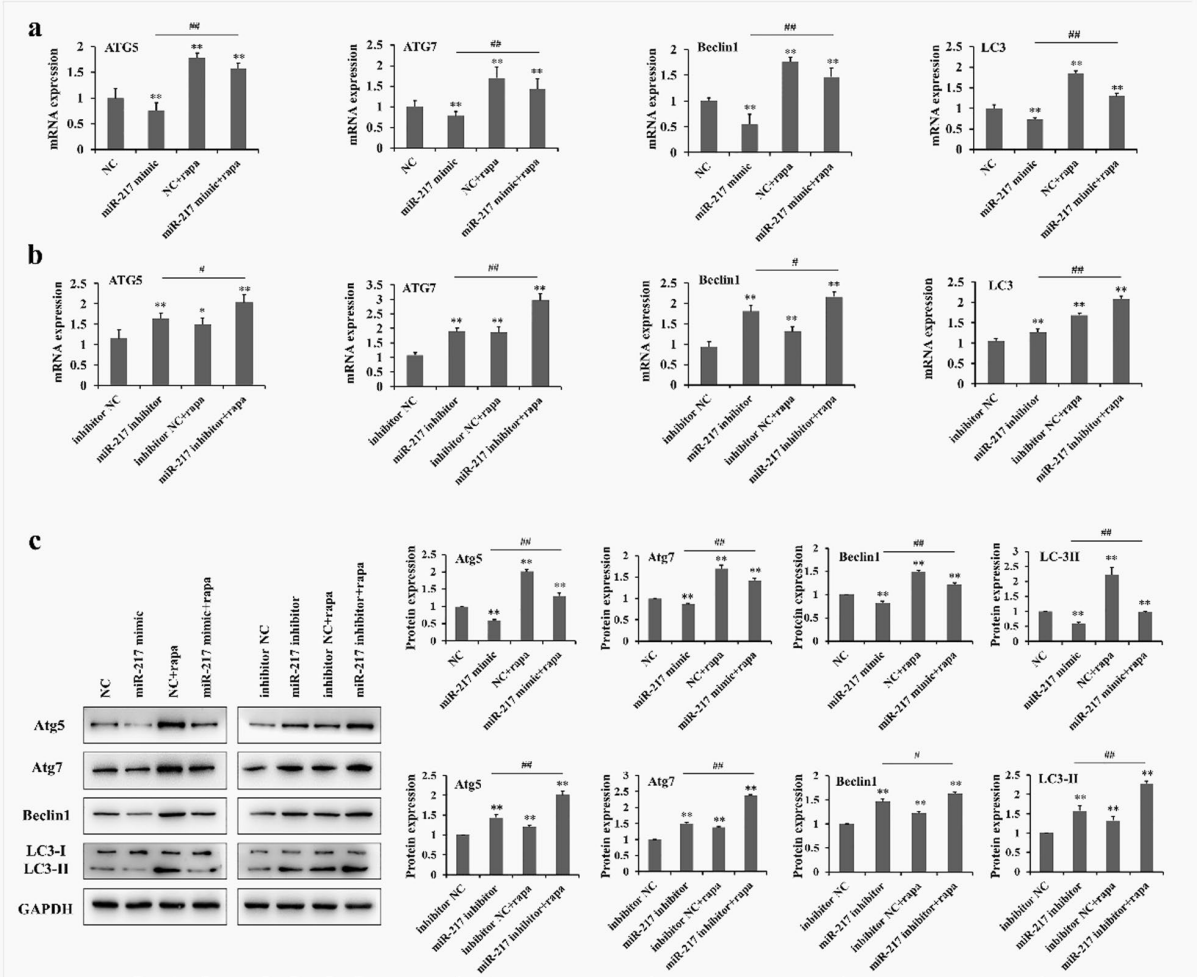
MiR-217 inhibits autophagy-related protein expression in GCT cells. **a**, **b** RT-PCR showing the changes in autophagy-related protein expression in GCT0404 cells following transfection with the miR-217 mimics or miR-217 inhibitor. **c** The autophagy-related proteins (Atg5, molecular weight: 55 kDa; Atg7, molecular weight: 78 kDa; Beclin-1, molecular weight: 60 kDa; LC3-I, molecular weight: 16 kDa; and LC3-II, molecular weight: 14 kDa) were detected in GCT0404 cells following transfection with miR-217 mimics or miR-217 inhibitor. **P < 0.01 versus control

Autophagy represents a key stress-response pathway that can suppress or promote tumorigenesis depending on the specific cellular context [14]. To investigate whether autophagy is involved in the process of miR-217 regulation of GCT cells, autophagy-related proteins were examined by RT-PCR and Western blots. Figure 3a shows that treatment with the miR-217 mimics significantly decreased the levels of ATG5, ATG7, Beclin-1, and LC-3 at mRNA expression in GCT0404 cells. Rapamycin (Rapa), a classic autophagy accelerant, was used to induce autophagy [26]. The RT-PCR results revealed that treatment with the miR-217 mimics could significantly inhibit the increased expression of ATG5, ATG7, Beclin-1, and LC-3 induced by Rapa in GCT0404 cells (Fig. 3a). In contrast, treatment with an miR-217 inhibitor significantly up-regulated the level of ATG5, ATG7, Beclin-1, and LC-3 expression in GCT0404 cells (Fig. 3b). Additionally, the level of ATG5, ATG7, Beclin-1, and LC-3 expression were further increased by treatment with an miR-217 inhibitor in Rapa-treated GCT0404 cells (Fig. 3b). Furthermore, for the expression of ATG5, ATG7 and Beclin-1, Western blot and RT-PCR results exhibited a similar tendency following miR-217 mimic or miR-217 inhibitor treatment (Fig. 3c). Importantly, the conversion of LC3 from LC3-I to LC3-II is a characteristic of autophagy [26]. Figure 3c indicates that the LC3-II/LC3-I ratio was significantly decreased in GCT0404 cells following exposure to the miR-217 mimic, whereas treatment with the miR-217 inhibitor was associated with an LC3-II/LC3-I ratio. As expected, the Western blot results demonstrated that Rapa treatment reversed the effects of miR-217 and enhanced the effects of the miR-217 inhibitor against autophagy-related proteins in GCT0404 cells (Fig. 3c).

**Fig. 4:**
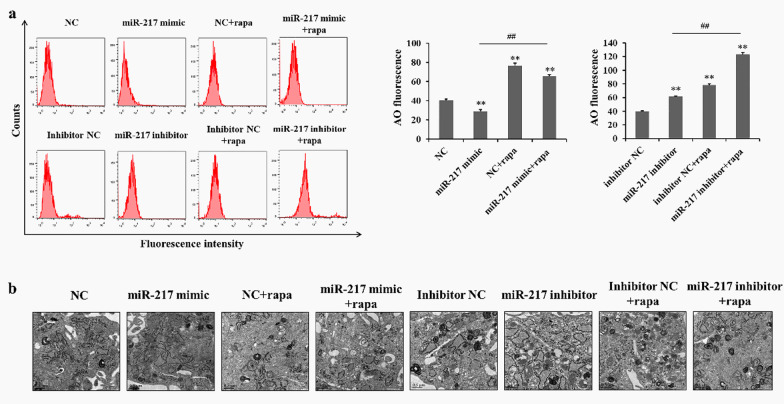
MiR-217 inhibits autophagosome/autolysosome formation in GCT cells. **a** GCT0404 cells were transfected with miR-217 mimics or an miR-217 inhibitor in the absence or presence of Rapa, and measured using AO staining; quantification (right). **b** Transmission electron microscopy images of GCT0404 cells were transfected with the miR-217 mimics or miR-217 inhibitor. Red arrowheads indicate autophagic vacuoles. **P < 0.01 versus control

AVOs, autophagy-related lysosomal structures, are monitored by labelling cells with AO [27]. Figure 4a shows that there was a significant decrease in AO fluorescence after 48 h transfection with the miR-217 mimic in GCT0404 cells. However, treatment with the miR-217 inhibitor significantly increased AO fluorescence in GCT0404 cells. Furthermore, the increase of AO fluorescence caused by Rapa could be reduced by treatment with the miR-217 mimic, but further enhanced by the miR-217 inhibitor in GCT0404 cells (Fig. 4a). Importantly, electron microscopy was used to identify a decreased number of autophagosomes/autolysosomes in miR-217 mimic-treated GCT0404 cells; however, the autophagosomes/autolysosomes were significantly increased by treatment with the miR-217 inhibitor (Fig. 4b). In addition, under the additional treatment of Rapa, changes of autophagosomes/autolysosomes were consistent with the Western blots and RT-PCR in GCT0404 cells (Fig. 4b). Therefore, the above data indicate that miR-217 treatment can inhibit autophagy in GCT cells.

### MiR-217 suppresses the growth of GCT cells xenografts in vivo

**Fig. 5:**
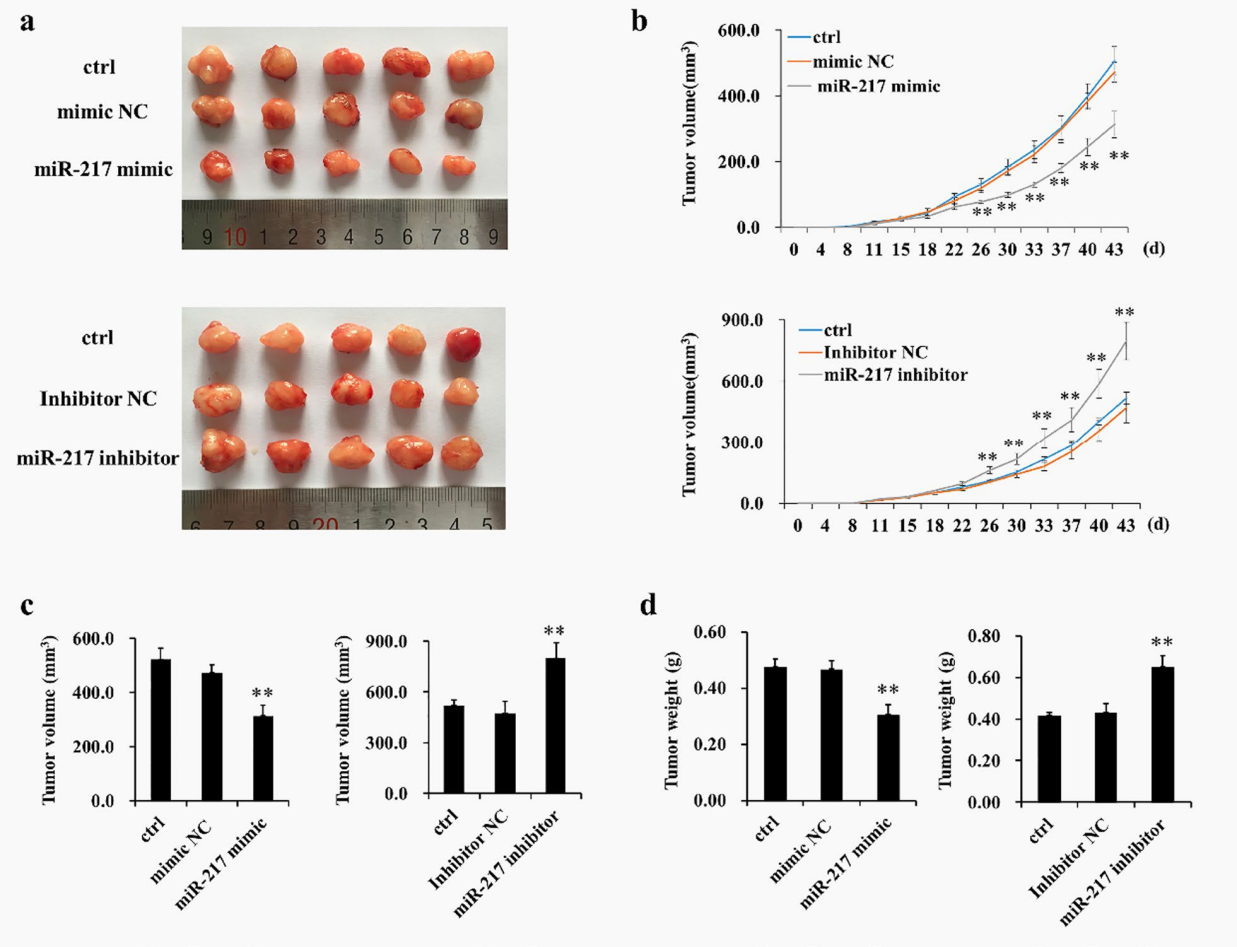
The anti-cancer effects of miR-217 in GCT cell-bearing mice. **a** Images of resected xenograft samples. **b** Average tumor volumes. **c** Average tumor weight. **P < 0.01 versus control

To confirm the effects of miR-217 on GCT in vivo, we examined the effects of miR-217 mimics and an inhibitor on tumor growth in a xenograft mouse model of GCT0404 cells. Figure 5a, shows that GCT0404 cell-bearing mice treated with miR-217 mimic exhibited attenuated tumor growth compared to mice in the model group. Importantly, the tumor volume and weight in the miR-217 mimic-treated group were obviously lower than those in the model group (Fig. 5b–d). In contrast, compared to the model group, treatment with the miR-217 inhibitor promoted tumor growth and increased the tumor volume and weight in GCT0404 cell-bearing mice (Fig. 5a–d).

### MiR-217 inhibits the growth of GCT cells by inducing autophagy

**Fig. 6:**
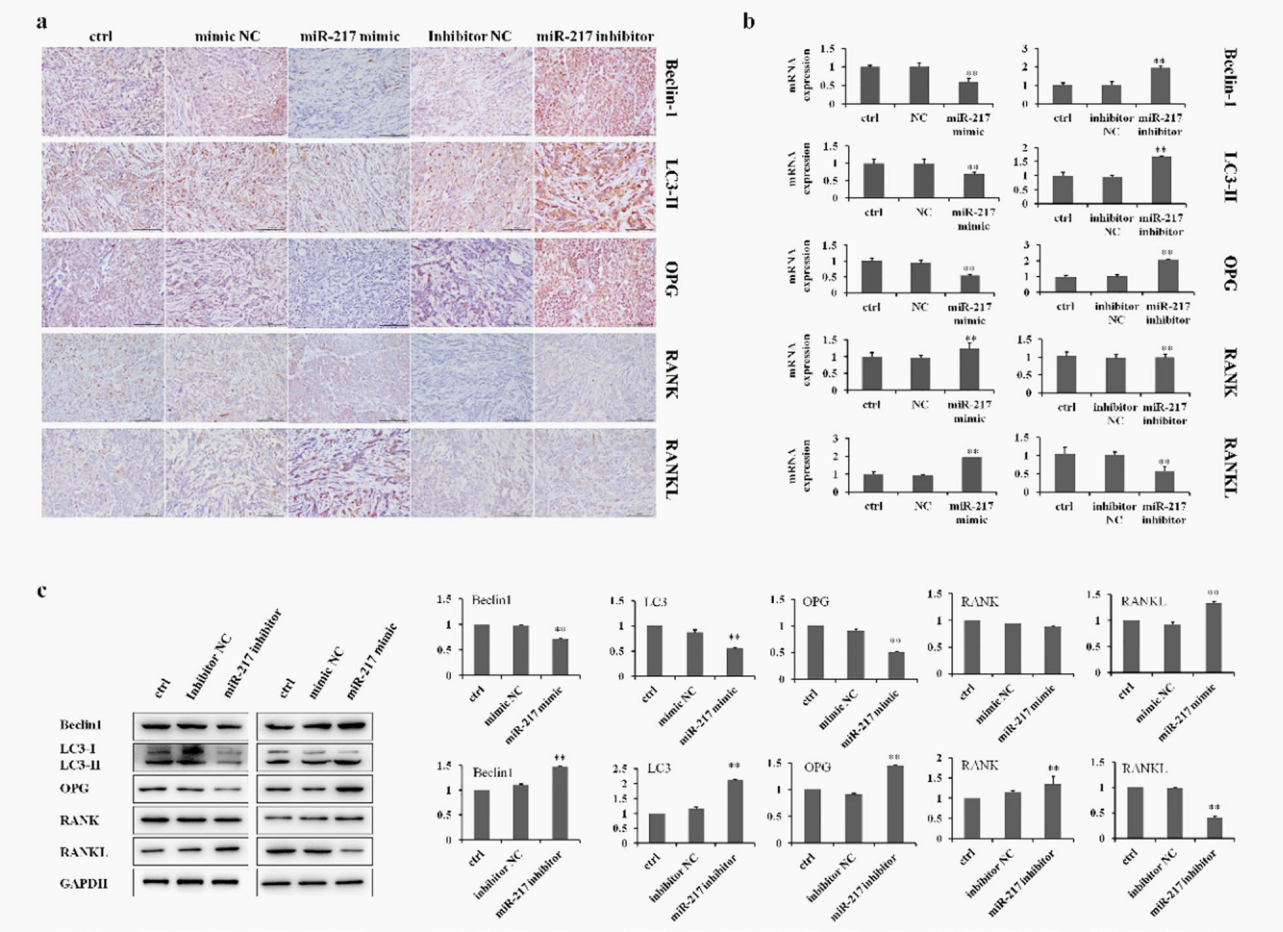
Protein expression in the OPG/RANKL/RANK signaling pathway by miR-217 in vivo. **a** Immunohistochemical staining of the OPG/RANKL/RANK signaling pathway-related protein expression in the tumor tissues (scale bar = 100 μm). **b** RT-PCR showing changes in the expression of proteins in tumors following transfection with miR-217 mimics or miR-217 inhibitor. **c** The level of protein (Beclin-1, molecular weight: 60 kDa; LC3-I, molecular weight: 16 kDa; LC3-II, molecular weight: 14 kDa; OPG, molecular weight: 46 kDa; RANK, molecular weight: 55 kDa; and RANKL, molecular weight: 35–45 kDa) expression was detected in the tumors by Western blot. **P < 0.01 versus control

The results from an immunohistochemistry assay showed that the level of Beclin-1, LC3-II, and OPG expression was significantly decreased by treatment with miR-217 mimics. Moreover, treatment with miR-217 mimics increased the level of RANKL expression in the GCT0404 cell tumors (Fig. 6a). Furthermore, Beclin-1, LC3-II, OPG, and RANKL expression were also examined by RT-PCR and Western blot. These results were consistent with the results of an immunohistochemistry assay in GCT0404 cell-bearing mice (Fig. 6b, c). It is important to note that treatment with the miR-217 inhibitor had the opposite effect compared to that of miR-217 mimic treatment (Fig. 6a–c). These results suggest that the OPG/RANKL/RANK signaling pathway is involved in the regulation of CGT cell tumor growth through miR-217-induced autophagy.

## Discussion

GCT of the bone is a benign lesion most often found in bone extremities associated with rapid growth [3, 4]. Although the primary clinical method for GCT treatment is surgery [5], half of patients with GCT exhibit local recurrence following primary surgical treatments [6]. Moreover, some patients can develop pulmonary metastases and spontaneous malignant transformation [2]. Therefore, there is an urgent need to perform in-depth studies of the molecular mechanisms of GCT to identify novel therapeutic targets and strategies.

MiRNAs are a group of highly conserved, endogenously expressed small non-coding RNA molecules [19]. Importantly, miRNAs play an essential role in the regulation of cellular proliferation, migration, and invasion by regulating gene expression and inhibiting protein translation [20–22]. MiR-217 plays an important role in different stages of bone formation [28] and studies indicate that it is highly related to both postmenopausal osteoporosis and senile osteoporosis [29, 30]. In the present study, we found that miR-217 overexpression could inhibit GCT0404 cell proliferation and colony formation. Other hand, treatment with an miR-217 inhibitor could promote GCT0404 cell growth in vitro. The expression of Ki67 was also significantly decreased in GCT0404 cells in response to miR-217 treatment. Furthermore, GCT0404 cells transfected with miR-217 exhibited poor tumorigenicity in nude mice. In contrast, treatment with an miR-217 inhibitor could remarkably promote cancer cells growth in GCT0404 cell-bearing mice. These results indicate that miR-217 exhibits potential anti-cancer effects and low toxicity in CGT cells both in vitro and in vivo. However, it is important to note that the efficiency of miRNA-based therapeutic molecules is dependent on delivery agents that can protect oligonucleotides from degradation, aggressively target pathological organs or tissues, and produce powerful therapeutic effects without adverse side effects [31]. Moreover, some research suggests that miRNA-based therapies may provide significant hope for the future, both for inhibiting and stimulating their expression. Limitations to overcome in using an appropriate delivery system include, poor in vivo stability, reduced biological distribution, and adverse side effects [32]. In addition, it is important to note that while miRNA can serve as a potential line of inquiry, another non-coding RNA, siRNA, may also contribute to the selection of future therapies. Studies have shown that siRNA can be used to identify molecules involved in various tendon repair processes. Several researchers have used siRNA to assess the impact of multiple transcription factors and their biological effects on various cancers. Gene identification, activation, and silencing can function as useful mechanisms and pharmacological targets for the production of novel specific drugs suitable for repairing tendon injuries. Thus, siRNA could be used to identify genes to target such future drugs and should be incorporated into future studies in addition to miRNA, a non-coding RNA [33].

OPG is a member of the tumor necrosis factor receptor superfamily and is widely expressed in a variety of tissues [9]. Recent studies have reported that OPG is functional only in the bone, and osteoblasts regulate the differentiation of osteoclasts via OPG and RANKL secretion and expression [10, 11]. Importantly, OPG can exhibit an inhibitory effect on RANK and its ligand, RANKL, and the OPG/RANKL/RANK signaling pathway is critical for adjusting bone metabolic balance [12, 13]. In this study, treatment with the miR-217 mimics significantly down-regulated the level of OPG expression and up-regulated the level of RANKL expression in GCT0404 cells. Furthermore, sh-OPG also increased the level of RANKL expression in GCT0404 cells; however, both treatment with the miR-217 inhibitor and OPG overexpression had opposing effects compared to that of the miR-217 mimics and sh-OPG in GCT0404 cells. Moreover, in GCT0404 cell-bearing mice, miR-217 mimics still displayed increased OPG expression and decreased RANKL expression, whereas the opposite was observed for the effects of the miR-217 inhibitor. Therefore, miR-217 exhibits anti-cancer activity against CGT cells through regulating the OPG/RANKL/RANK signaling pathway both in vitro and in vivo.

Autophagy is an evolutionarily conserved catabolic process that involves maintaining the quality control of proteins and organelles, as well as energy homeostasis [14, 15]. Additionally, autophagy is up-regulated in multiple cancer cells and promotes cellular survival under conditions of stress; thus, autophagy may be considered a potential therapeutic target for cancer [16–18]. As expected, miR-217 could significantly decrease the expression of ATG5, ATG7, Beclin-1, and LC-3 at both the protein and mRNA level. In addition, the conversion of LC3 from LC3-I to LC3-II was identified as a marker involved in the formation of autophagosomal vacuoles [26]. In the present study, the fluorescence intensity of AO exhibited an obvious decline following miR-217 treatment in GCT0404 cells. Importantly, the number of double-membrane autophagosomes was also decreased under miR-217 treatment in GCT0404 cells. However, treatment with the miR-217 inhibitor led to the opposite results of autophagy-related protein expression in GCT0404 cells. In addition, miR-217 mimics could inhibit Rapa-induced autophagy, and treatment with the miR-217 inhibitor further enhanced the effects of Rapa. Taken together, miR-217 exhibits anti-cancer effects against GCT cells through inducing autophagy both in vitro and in vivo.

## Conclusion

In summary, our results revealed that miR-217 may function as a tumor suppressor in GCT cells. In particular, autophagy induced by the overexpression of miR-217 could suppress GCT cell proliferation and tumorigenesis through suppressing the OPG/RANKL/RANK signaling pathway both in vitro and in vivo. Together, these findings suggest that the down-regulated miR-217 can contribute to GCT progression. Therefore, miR-217 might exhibit therapeutic potential for GCT treatment.

## Data Availability

The analyzed datasets generated during the study are available from the corresponding author on reasonable request.
